# MAFG-AS1 aggravates the progression of pancreatic cancer by sponging miR-3196 to boost NFIX

**DOI:** 10.1186/s12935-020-01669-y

**Published:** 2020-12-09

**Authors:** Liqing Ye, Weijian Feng, Hanqin Weng, Chongde Yuan, Jia Liu, Zaiguo Wang

**Affiliations:** Department of Hepato-Billiary Surgery, Dongguan People’s Hospital, Southern Medical University, Guangming Road, Dongcheng District, Dongguan, 523059 Guangdong China

**Keywords:** MAFG-AS1, miR-3196, NFIX, Pancreatic cancer

## Abstract

**Background:**

A host of researches have demonstrated the regulation of long non-coding RNAs (lncRNAs) in the progression of pancreatic cancers (PC). In this study, our main task was to analyze the function of MAF bZIP transcription factor G antisense RNA 1 (MAFG-AS1) in PC.

**Methods:**

RT-qPCR measured gene expression. Functional experiments, including EdU assay, flow cytometry analysis, TUNEL assay and transwell assay, assessed the biological changes of PC cells. RNA pull down assay, luciferase reporter assay and RIP assay verified the interaction between RNAs.

**Results:**

MAFG-AS1 was lowly expressed in normal pancreatic samples but up-regulated in PC tissues and cell lines. Besides, MAFG-AS1 silence suppressed cell proliferation and migration whereas promoted cell apoptosis in PC. Mechanism assays verified that miR-3196 could bind with MAFG-AS1. Moreover, miR-3196 was discovered to be lowly expressed in PC cell lines, and its overexpression inhibited PC cell growth and migration. Importantly, nuclear factor I X (NFIX), overexpressed in PC cell lines, was validated to be positively modulated by MAFG-AS1 through absorbing miR-3196. Moreover, overexpression of NFIX could countervail the restraining effects of MAFG-AS1 knockdown on the growth and migration of PC cells.

**Conclusion:**

MAFG-AS1 had an oncogenic function in the progression of PC via regulating miR-3196/NFIX pathway, and decreasing MAFG-AS1 expression could attenuate PC progression.

## Background

Pancreatic cancer (PC) is a common tumor in the digestive system around the globe, characterized by intensive migration and high invasion [1]. Though great achievements have been made in comprehensive therapies of cancers, the morbidity and mortality of PC are still increasing, which makes it a great threat to human life and health [2]. The prognosis of PC is rather dismal in the past decade [3]. Surgery is a routine treatment for PC patients, but the recurrence rate is extremely high due to frequent metastasis [4]. The detailed cause of PC is still a riddle. Hence, it is necessary to study the pathological feature of PC and figure out the underlying mechanism to find out effective therapeutic ways.

Long non-coding RNAs (lncRNAs) are widely identified to modulate biological progression in multiple cancers. For instance, lncRNA PCAT-1 accelerated NSCLC progression by targeting miR-149-5p to regulate LRIG2 [5]. MALAT1 promoted the progression of ovarian cancer through modulating miR-506 and iASPP [6]. Lnc-ATB facilitated cell proliferation in gastric cancer by miR-141-3p/TGFβ2 signaling [7]. MAF bZIP transcription factor G antisense RNA 1 (MAFG-AS1) is a new lncRNA that has been discovered to expedite cell proliferation and invasion in colorectal cancer through targeting miR-147b/NDUFA4 [8]. Nevertheless, the function of MAFG-AS1 has not been elucidated in PC.

Increasing essays certified that lncRNAs function as competing endogenous RNAs (ceRNAs) of messenger RNAs (mRNAs) to bind with microRNAs (miRNAs) so that mRNAs could be released from the binding of miRNAs. This regulation mode is known as the ceRNA mechanism [9]. For instance, LINC00511 boosted the progression of breast cancer through targeting miR-185-3p/E2F1/Nanog [10]. FER1L4 inhibited cell migration in osteosarcoma via sponging miR-18a-5p to modulate PTEN [11]. XIST facilitated the growth of thyroid cancer cells through sequestering miR-34a to modulate MET-PI3K-AKT pathway [12]. This study made investigations on whether MAFG-AS1 could be a ceRNA in PC.

The current study analyzed the role of MAFG-AS1 in the progression of PC. Moreover, we also assessed the modulatory mechanism of MAFG-AS1 in PC.

## Methods

### Sample collection

The PC tissues and paired non-cancerous samples were acquired from 65 PC patients who underwent surgery in Dongguan People’s Hospital Hospital. Samples were processed with liquid nitrogen for quick-frozen, and then maintained at -80℃. Patients who received other treatment before operation were excluded. All the relevant patients signed the informed consents prior to surgery. This study was conducted with the approval of the Ethics Committee of Dongguan People’s Hospital numbered (DGP2019-076).

### Cell culture

Four kinds of PC cell lines (Capan 1, CFPAC-1, SW1990 and PANC-1) were procured from ATCC (Manassas, VA, USA). Human normal pancreatic cancer cell line HPC-Y5 was purchased from China Center for Type Culture Collection (CCTCC; Wuhan, China) and cultured in Eagle’s Minimum Essential Medium. Capan 1 and CFPAC-1 cells were kept in Iscove’s Modified Dulbecco’s Medium, SW1990 cells were maintained in Leibovitz’s L-15 Medium, and PANC-1 cells were cultivated in Dulbecco’s Modified Eagle’s Medium (GIBCO, Rockville, MD, USA). 10% fetal bovine serum (FBS; GIBCO), 100U/ml penicillin and 100 μg/ml streptomycin (GIBCO) were the medium supplements.

### Plasmids and transfection

Two short hairpin RNAs (sh-MAFG-AS1#1 and sh-MAFG-AS1#2) and non-targeting plasmids (sh-NC) were obtained from Genechem (Shanghai, China). NC mimics and miR-3196 mimics were bought from Ribobio (Guangzhou, China), so were NC inhibitor and miR-3196 inhibitor. Whole cDNA sequence of NFIX was cloned into pcDNA3.1 vector (Invitrogen, Carlsbad, CA, USA) to produce pcDNA3.1-NFIX, with the empty vector as negative control. All transfections were completed with Lipofectamine 3000 (Invitrogen) as per the manufacturer’s guidelines.

### RT-qPCR

TRIzol reagent (Invitrogen) was used to extract total RNA from cultured cells, and total RNA was reversely transcribed into cDNA by using PrimeScript™ RT reagent kit (TaKaRa, Shiga, Japan). RT-qPCR analyses were carried out through using SYBR green Supermix (Thermo Fisher, Waltham, MA, USA) according to the manufacturers’ instructions. Relative gene expression was calculated by 2^−ΔΔCt^ method. GAPDH and U6 acted as normalized genes. The experiment was repeated at least three times. The sequences of primers were listed in Table 1.

**Table 1:** List of primer sequences

Primer	Sequence (5′-3′)
MAFG-AS1-forward (F)	GAGAGCTGAAGGTGTTCCGT
MAFG-AS1-reverse (R)	GGAGTGAGAGGGATGCTTGG
miR-3196-F	CGGGGCGGCAGGGGCCTC
miR-3196-R	CTCAACTGGTGTCGTGGA
PIP5K1C-F	CTCCATTGCCACGACTCTGT
PIP5K1C-R	TGTCCAGACGACTGTGTGC
NFIX-F	TACCAGCAGCGTGTGATGAG
NFIX-R	TGATGGTCAGCACGAAGTCC
SYNGAP1-F	TCCGAAGTGCTGACCATGAC
SYNGAP1-R	TTTACTGCCCGCTGCAGATT
GAPDH-F	GGAGCGAGATCCCTCCAAAAT
GAPDH-R	GGCTGTTGTCATACTTCTCATGG
U6-F	CTCGCTTCGGCAGCACA
U6-R	AACGCTTCACGAATTTGCGT

### EdU

EdU assay was completed with BeyoClick™ EdU Cell Proliferation Kit (Beyotime, Shanghai, China) based on the user guide. Cells were planted on 96-well plates (1 × 10^4^ cells/well) and fixed by 4% paraformaldehyde for 30 min. 0.5% Troxin X-100 was utilized to permeate cells for 10 min. Results were acquired using fluorescent microscope (Leica, Wetzlar, Germany). The experiment was repeated at least three times.

### Flow cytometry analysis

Cells (1 × 10^6^) were first planted into 6-well plates for 48 h and then washed twice with PBS. After resuspension, the precooled 70% ethanol was employed to fix cells at 4 ℃ for 1 h. Annexin V-FITC/PI double staining kit (Invitrogen) was utilized for detecting apoptotic cells. At last, samples were analyzed with flow cytometer (BD Biosciences, Franklin Lakes, NJ, USA). The experiment was repeated at least three times.

### TUNEL staining assay

Cells after transfections were fixed by 4% paraformaldehyde at 37℃ in darkness. Later, cells were permeated with 0.1% Triton x-100 for 5 min. Then PBS was applied to rinse cells thrice. In line with the manufacturer’s instructions, cell apoptosis was assessed using One-Step TUNEL Apoptosis Assay Kit (Beyotime). DAPI was taken to stain nuclei. Apoptotic cells were observed through fluorescence microscope (Leica). The experiment was repeated at least three times.

### Transwell assays

Cell migration assay was conducted by transwell chamber (Corning Incorporated, Corning, NY, USA), in line with the standard protocol. Cells were added into the upper chamber, and the lower chamber was supplemented with complete medium. After 24 h of incubation, the migrating cells were assessed in five random fields using optical microscope (Olympus). The experiment was repeated at least three times.

### Luciferase reporter gene assay

For luciferase reporter gene assay, cells were co-transfected with indicated pmirGLO vectors (Promega, Madison, WI, USA) which were loaded with the full-length MAFG-AS1 or NFIX 3′-UTR sequence containing wild-type or mutant miR-3196 binding sites, in the presence of miR-3196 mimics or NC mimics. After 48 h of co-transfection, relative luciferase activity was monitored using Luciferase Reporter Assay System (Promega). The experiment was repeated at least three times.

### FISH

RNA FISH KIT (Ribobio) was applied to confirm the subcellular location of MAFG-AS1. The RNA FISH probe mix was synthesized and created by RiboBio. The air-dried cells were treated with specific probes and hybridization buffer. DAPI (Beyotime) was used to stain cell nuclei. The fluorescent detection was completed by fluorescence microscope (Olympus). The experiment was repeated at least three times.

### Subcellular fractionation

PARIS™ Kit (Invitrogen) was utilized to isolate cytoplasmic and nuclear RNAs from SW1990 and PANC-1 cells, in light of the provided instruction. Briefly, cells were lysed with cell fractionation buffer, followed by the separation of cytoplasmic and nuclear fractions by centrifuge. U6 was used as the nuclear control and GAPDH as the cytoplasmic control. Expression levels of RNAs were analyzed by RT-qPCR. The experiment was repeated at least three times.

### In situ hybridization (ISH)

ISH assay was implemented as per the user guide (Boster Bio-Technology Company). Briefly, the paraffin-embedded sample sections with 4 μm thickness were subjected to dewaxing via xylene, rehydration by diluted reagent grade ethanol, and digestion with protease K. Afterwards, the sections were processed with specific MAFG-AS1 probes labeled with digoxigenin at 37 ℃ overnight, followed by 30 min of the treatment with HRP-conjugated anti-digoxin antibody at 25 ℃. Diaminobenzidine was the HRP substrate for staining. The images were captured by an optical microscope (Olympus). The experiment was repeated at least three times.

### RNA immunoprecipitation (RIP)

RIP assay was carried out using the Magna RIP™ RNA-Binding Protein Immunoprecipitation Kit (Millipore, Billerica, MA, USA). The prepared cells were lysed in RIP lysis buffer, and then treated with RIP buffer containing magnetic beads conjugated with human Ago2 antibody or normal IgG antibody. Afterwards, the retrieved RNA was assayed by RT-qPCR. The experiment was repeated at least three times.

### Pull down assay

The interaction between RNAs was examined by RNA pull down assay by use of Pierce Magnetic RNA–Protein Pull-Down Kit (Thermo Fisher), as guided by the provider. After collecting the extracts from cells, the magnetic beads were added to capture the RNA-RNA complex. At length, RNAs in the complex were eluted and finally analyzed by RT-qPCR. The experiment was repeated at least three times.

### Statistical analysis

Bio-repeats were conducted thrice, and all results were exhibited as the mean ± SD. Data were analyzed by Student’s t-test or one-way analysis of variance (ANOVA) using GraphPad PRISM 6 (GraphPad, San Diego, CA, USA). Differences were regarded as statistically significant with *P* < 0.05.

## Results

### MAFG-AS1 was aberrantly upregulated in PC cells and promoted the progression of PC

**Fig. 1:**
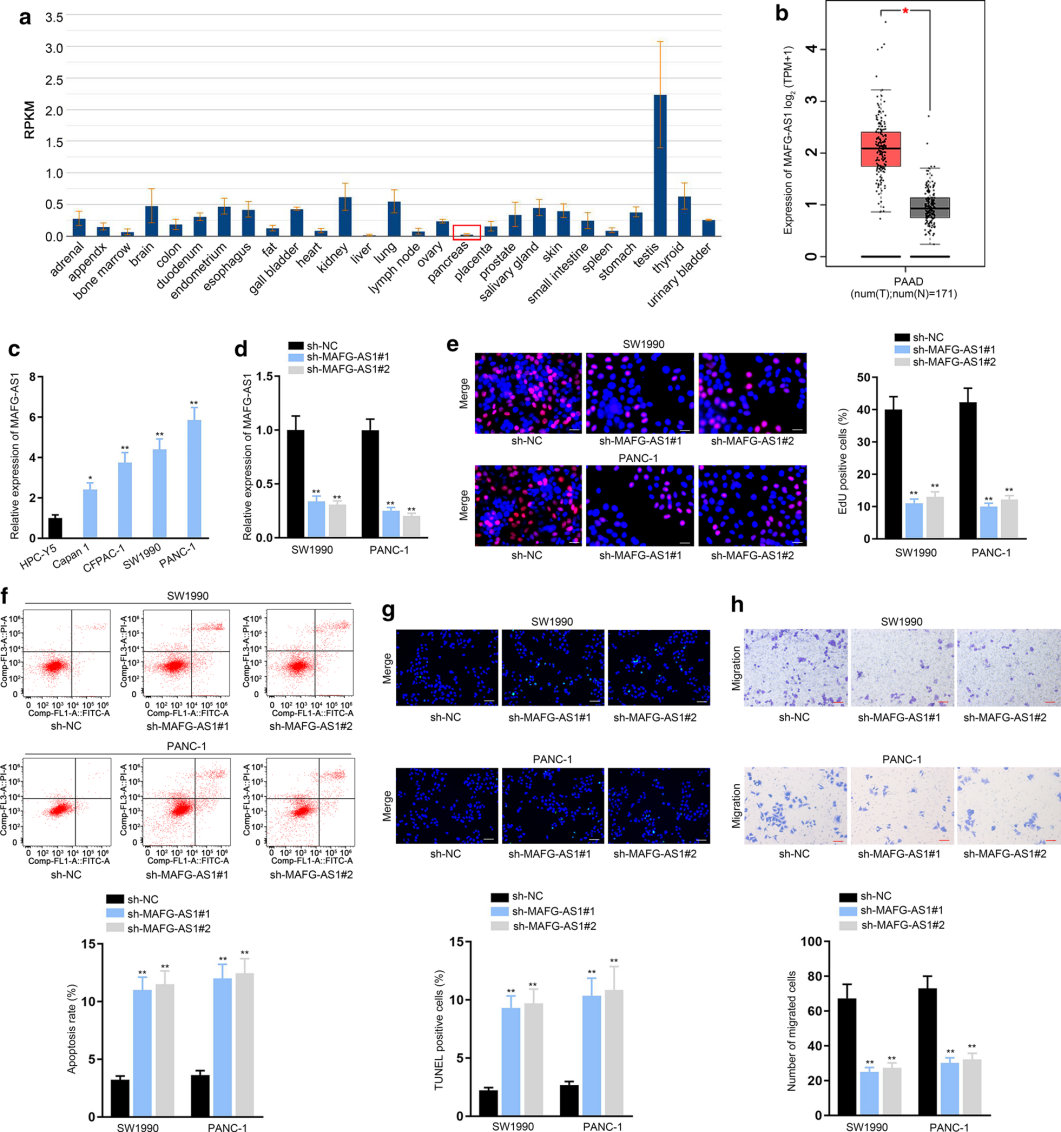
MAFG-AS1 was aberrantly upregulated in PC cells and its silence hindered the progression of PC. a NCBI data disclosed the expression pattern of MAFG-AS1 in normal tissue samples. **b** GEPIA data of MAFG-AS1 expression in PAAD tissues and normal pancreatic tissues. **c** RT-qPCR assessed MAFG-AS1 expression in PC cell lines (Capan 1, CFPAC-1, SW1990 and PANC-1) and normal pancreatic cell line (HPC-Y5). **d** Knockdown efficiency of sh-MAFG-AS1#1/2 was examined by RT-qPCR in SW1990 and PANC-1 cells. **e** EdU assays evaluated the proliferation of cells transfected with sh-MAFG-AS1#1/2. Scale bar = 100 μm. **f**, **g** The impact of MAFG-AS1 suppression on cell apoptosis was evaluated by flow cytometry analysis and TUNEL assays (scale bar = 200 μm). **h** Cell migration in different groups was appraised through transwell assays (scale bar = 200 μm). *P < 0.05, **P < 0.01

Firstly, we searched gene expression in normal tissues via NCBI (https://www.ncbi.nlm.nih.gov/gene/92659). Results validated that MAFG-AS1 was lowly expressed in normal pancreatic samples (Fig. 1a). Next, according to GEPIA (http://gepia.cancer-pku.cn/) database, MAFG-AS1 was highly expressed in PAAD (pancreatic adenocarcinoma) tissues compared with normal pancreatic tissues (Fig. 1b, *P < 0.05). Moreover, we also verified the upregulation of MAFG-AS1 in PC samples relative to adjacent non-tumor ones (Additional file 1: Fig. S1a, **P < 0.01). Data of ISH further proved the high positivity of MAFG-AS1 in PC tissues (Additional file 1: Fig. S1b). Similarly, MAFG-AS1 expression was higher in PC cell lines (Capan 1, CFPAC-1, SW1990 and PANC-1) than normal pancreatic cell line (HPC-Y5) (Fig. 1c, *P < 0.05, **P < 0.01). SW1990 and PANC-1 cells were used for following assays because they had higher MAFG-AS1 expression. Then, the impact of MAFG-AS1 on the function of PC cells was focused on. Hence, sh-MAFG-AS1#1/2 were transfected into SW1990 and PANC-1 cells to reduce the expression of MAFG-AS1 (Fig. 1d, **P < 0.01). Data of EdU assays revealed that the proliferation of PC cells was decreased by MAFG-AS1 knockdown (Fig. 1e, **P < 0.01). In contrast, the apoptosis rate was augmented in MAFG-AS1-depleted PC cells (Fig. 1f, g, **P < 0.01). Meanwhile, the results of transwell assays evidenced that cell migration was inhibited by the down-regulation of MAFG-AS1 (Fig. 1h, **P < 0.01). Altogether, MAFG-AS1 was aberrantly upregulated in PC cells and promoted the progression of PC.

### MiR-3196 bound with MAFG-AS1

**Fig. 2:**
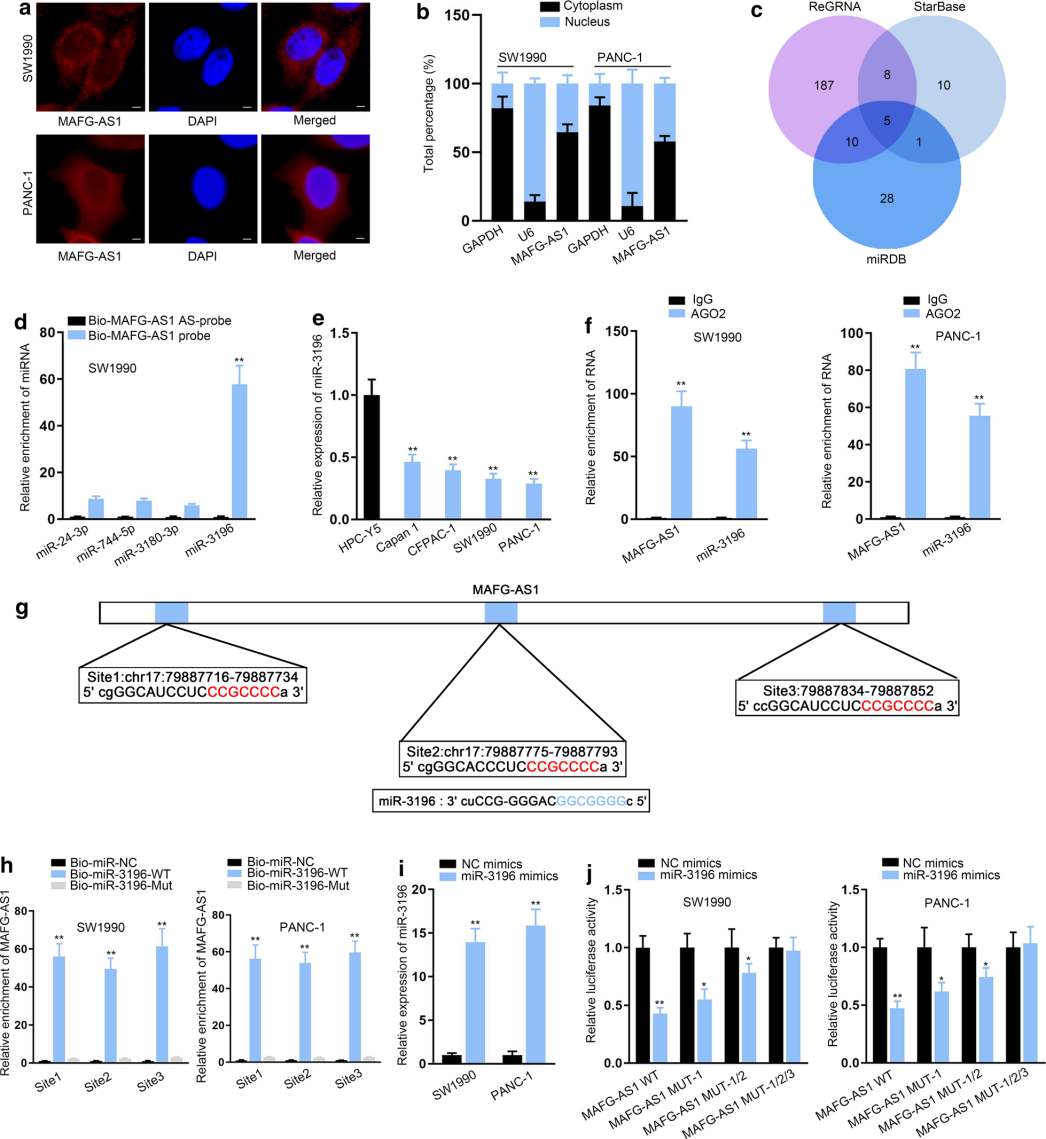
MiR-3196 bound with MAFG-AS1. **a**, **b** FISH (scale bar = 20 μm) and nucleus cytoplasm fractionation assays located MAFG-AS1 in cytoplasm. **c** Venn diagram showed 4 mutual miRNAs interacting with MAFG-AS1 that were found after searching starBase, ReGRNA and miRDB databases. **d** RNA pull down assays detected the interaction of MAFG-AS1 with the selected 4 miRNAs. **e** MiR-3196 expression was measured in PC cell lines via RT-qPCR. **f** RIP assays exhibited the interaction between MAFG-AS1 and miR-3196. **g** Binding sites between miR-3196 and MAFG-AS1 were shown. **h** RNA pull down examined the binding sites responsible for the interaction between miR-3196 and MAFG-AS1. **i** RT-qPCR detectedmiR-3196 expression in PC cells transfected with miR-3196 mimics or NC mimics. **j** Luciferase reporter assays appraised the binding between miR-3196 and MAFG-AS1. **P < 0.01

Then, we analyzed possible regulatory mechanism of MAFG-AS1 in PC. Data of FISH assay and nucleus cytoplasm fractionation showed that MAFG-AS1 was accumulated in the cytoplasm of PC cells (Fig. 2a, b), indicating the probable ceRNA feature of MAFG-AS1. Hence, we started to find out the downstream of MAFG-AS1. Venn diagram showed 4 mutual miRNAs binding with MAFG-AS1 from starBase, ReGRNA and miRDB tools (Fig. 2c). RNA pull down assay results depicted miR-3196 was more easily pulled down by biotinylated MAFG-AS1 (Fig. 2d, **P < 0.01). Therefore, miR-3196 was selected out. RT-qPCR measured miR-3196 expression and proved its downregulation in PC cell lines (Fig. 2e, **P < 0.01). Data of RIP assays exhibited that MAFG-AS1 and miR-3196 were enriched by Ago2 antibody but not by IgG antibody (Fig. 2f, **P < 0.01). We found there were 3 binding sites between miR-3196 and MAFG-AS1 (Fig. 2g). Additionally, RNA pull down data displayed that miR-3196 bound with MAFG-AS1 at all these three sites (Fig. 2h, **P < 0.01). Then, miR-3196 mimics were transfected into cells to increase miR-3196 expression (Fig. 2i, **P < 0.01). The outcomes of luciferase reporter assays presented that up-regulation of miR-3196 suppressed the luciferase activity of MAFG-AS1-WT and also had a certain effect on that of site 1- or site 1/2-mutated MAFG-AS1, whereas did not affect the activity of MAFG-AS1 with mutant three sites (Fig. 2j, *P < 0.05, **P < 0.01). To sum up, miR-3196 was the downstream of MAFG-AS1.

### MiR-3196 hindered the progression of PC

**Fig. 3:**
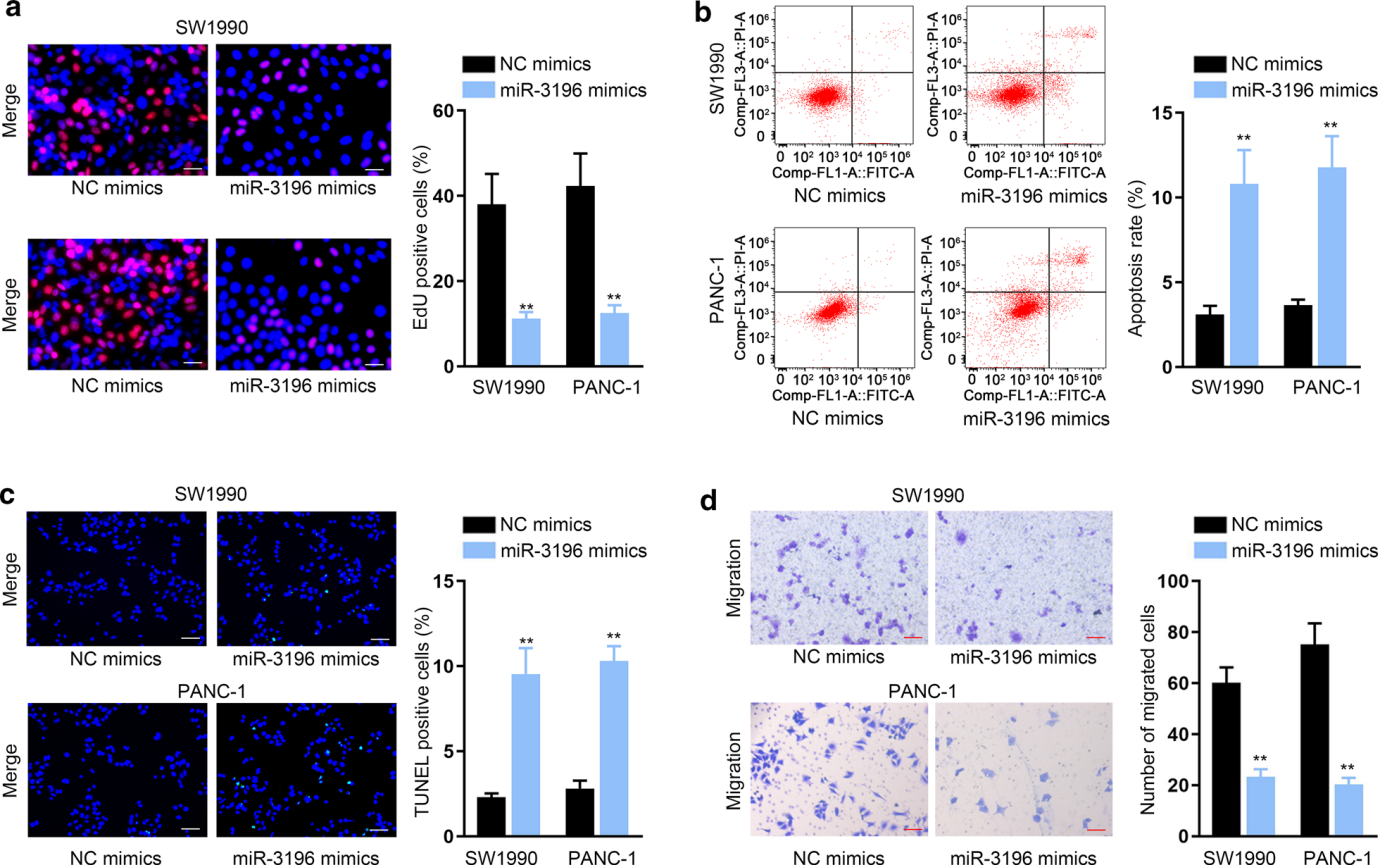
MiR-3196 hindered the progression of PC. **a**–**d** EdU assay (scale bar = 100 μm) (**a**), flow cytometry (**b**), TUNEL assay (scale bar = 200 μm) (**c**) and transwell assay (scale bar = 200 μm) (**d**) examined the effects of miR-3196 upregulation on PC cell proliferation, apoptosis and migration. **P < 0.01

The impacts of miR-3196 on the biological behaviors of PC cells were investigated as well. Data of EdU assays exhibited that overexpression of miR-3196 reduced the ability of PC cells to proliferate (Fig. 3a, **P < 0.01). Conversely, the apoptosis rate was increased when miR-3196 was up-regulated in PC cells (Fig. 3b, c, **P < 0.01). Meanwhile, cell migratory capacity was repressed under miR-3196 up-regulation (Fig. 3d, **P < 0.01). To sum up, miR-3196 inhibited the growth and migration of PC cells.

### MiR-3196 bound with NFIX 3′ UTR

**Fig. 4:**
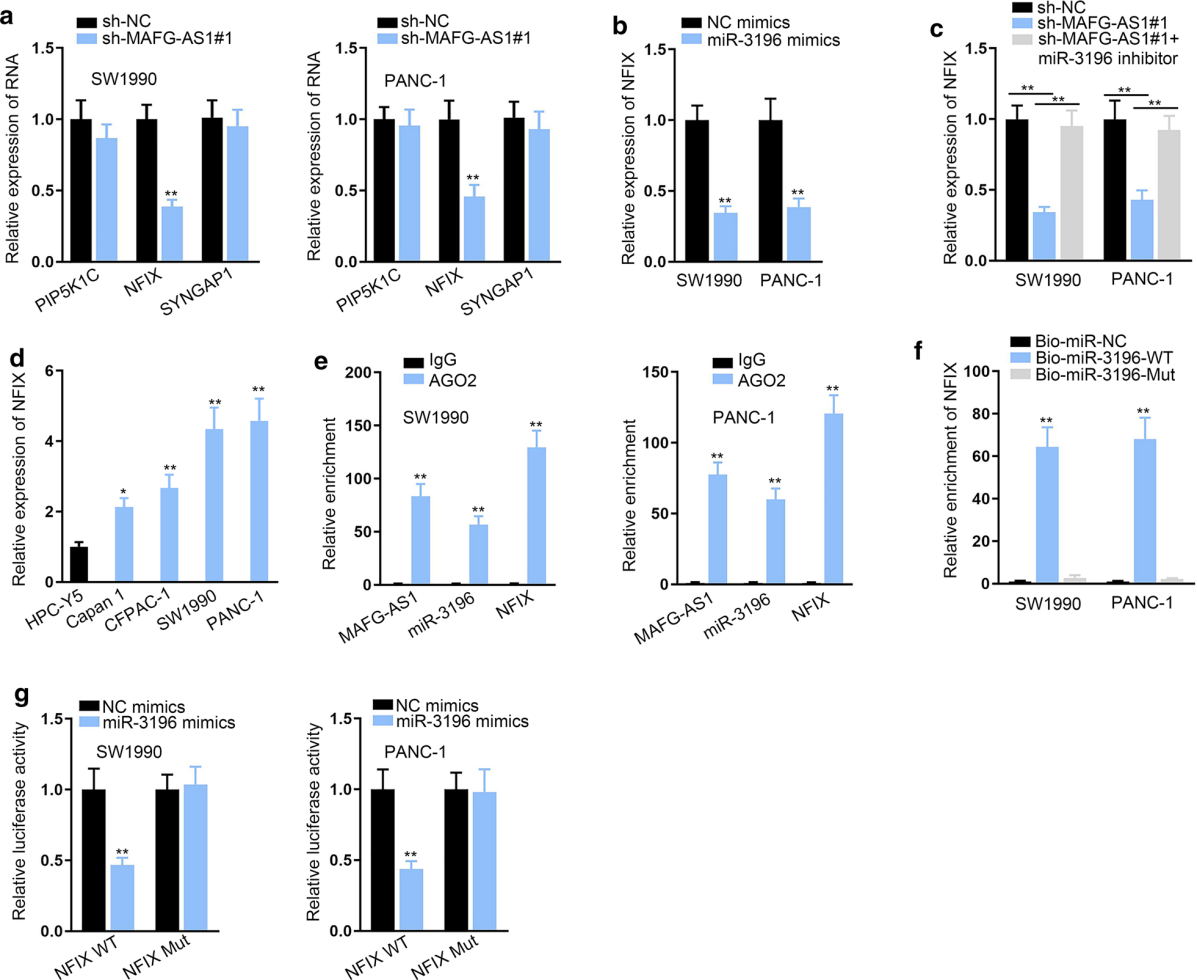
MiR-3196 bound with NFIX 3′ UTR. **a** The expression of three mRNAs was assessed by RT-qPCR in cells with or without MAFG-AS1 inhibition. **b** NFIX expression was detected by RT-qPCR in cells with miR-3196 mimics or NC mimics. **c** NFIX expression was measured by RT-qPCR in cells transfected with sh-NC, sh-MAFG-AS1#1 or sh-MAFG-AS1#1 plus miR-3196 inhibitor. **d** NFIX expression was detected in PC cell lines through RT-qPCR. **e** RIP assay results showed that MAFG-AS1, miR-3196 and NFIX were enriched by Ago2 antibody. **f** RNA pull down examined miR-3196 bound with NFIX. **g** Luciferase reporter assays validated the binding between miR-3196 and NFIX. *P < 0.05, **P < 0.01

For the sake of further investigating the downstream effector of MAFG-AS1/miR-3196 axis in PC, we searched starBase with specific conditions (Degradome-Data ≥ 1, Pan-Cancer ≥ 4) and found out 3 probable targets of miR-3196 in microT database. Furthermore, we found that only the expression of nuclear factor I X (NFIX) was lessened while PIP5K1C and SYNGAP1 expressions showed no apparent changes in MAFG-AS1-silenced PC cells (Fig. 4a, **P < 0.01). Likewise, NFIX expression was diminished by overexpressed miR-3196 (Fig. 4b, **P < 0.01). Interestingly, we also found that the reduced NFIX expression by MAFG-AS1 down-regulation was then recovered in face of miR-3196 inhibition (Fig. 4c, **P < 0.01). Data of RT-qPCR exhibited NFIX expression was significantly high in PC cells (Fig. 4d, *P < 0.05, **P < 0.01). RIP assay results disclosed that MAFG-AS1, miR-3196 and NFIX were precipitated by Ago2 antibody not IgG antibody (Fig. 4e, **P < 0.01). Moreover, data of RNA pull down assays validated that miR-3196 bound with NFIX (Fig. 4f, **P < 0.01). The results of luciferase reporter assays further certified that up-regulation of miR-3196 restrained the luciferase activity of NFIX-WT but failed to affect that of NFIX-Mut (Fig. 4g, ^**^P < 0.01). Taken together, NFIX was the downstream of MAFG-AS1/miR-3196 signaling in PC.

### MAFG-AS1 promoted the progression of PC via elevating NFIX

**Fig. 5:**
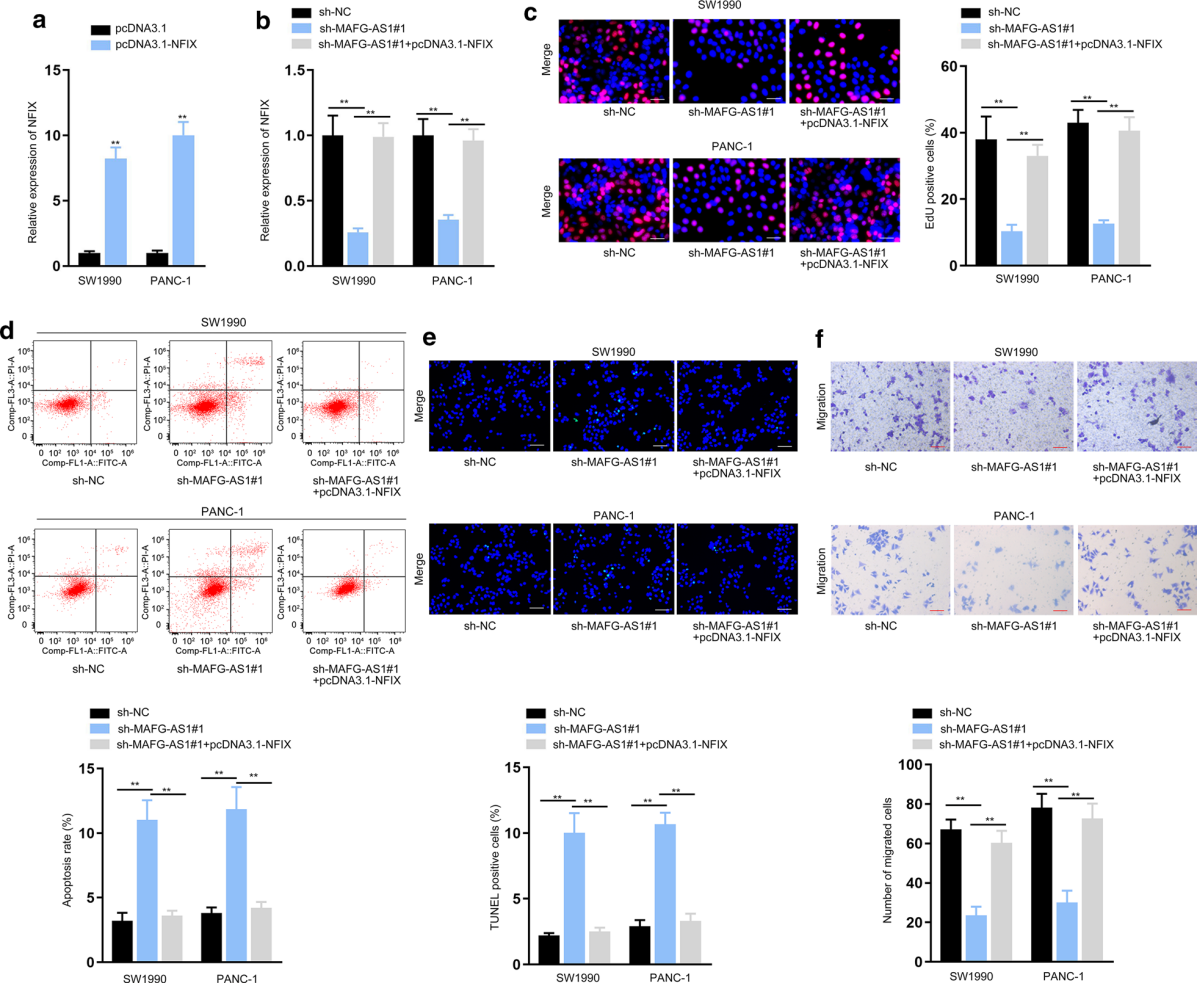
MAFG-AS1 promoted the malignant course of PC cells via elevating NFIX. **a** RT-qPCR proved the augmented NFIX expression in PC cells transfected with pcDNA3.1-NFIX. **b** NFIX expression was detected by RT-qPCR in cells transfected with sh-NC, sh-MAFG-AS1#1 or sh-MAFG-AS1#1 plus pcDNA3.1-NFIX. **c**–**f** EdU assay (scale bar = 200 μm) (**c**), flow cytometry (**d**), TUNEL assay (scale bar = 200 μm) **e** and transwell assay (scale bar = 200 μm) **f** examined the proliferation, apoptosis and migration of PC cells under different conditions. **P < 0.01

Then, rescue assays were performed to validate whether MAFG-AS1 modulated NFIX to influence the progression of PC. In this case, NFIX expression was enhanced in PC cells by the transfection of pcDNA3.1-NFIX (Fig. 5a, **P < 0.01). Also, it was proved that the falling trend of NFIX expression induced by MAFG-AS1 depletion was then recovered under the co-transfection of pcDNA3.1-NFIX (Fig. 5b, **P < 0.01). Seen from the results of EdU assays, PC cell proliferation was suppressed by MAFG-AS1 silence, while such descending tendency was neutralized after NFIX recovery (Fig. 5c, **P < 0.01). Besides, the apoptosis of PC cells accelerated by MAFG-AS1 knockdown was reversed by the overexpression of NFIX (Fig. 5d, e, **P < 0.01). Likewise, the falling trend of cell migratory ability imposed by down-regulated MAFGAS1 was offset by up-regulated NFIX (Fig. 5f, **P < 0.01). In conclusion, MAFG-AS1 facilitated the oncogenic phenotypes of PC cells by enhancing NFIX expression.

## Discussion

Mounting essays have illustrated that lncRNAs have crucial functions in the biological progression of various cancers. For example, lncRNAs FTX facilitated the progression of gastric cancer via sponging miR-144 to elevate ZFX [13]. EPIC1 was reported to interact with YAP1 to facilitate the growth of PC cells [14]. DILC had oncogenic contributions to the progression of gallbladder carcinoma [15]. In previous studies, MAFG-AS1 has been introduced to promote the progression of lung adenocarcinoma [16] and breast carcinoma [17]. Presently, MAFG-AS1 was first detected to be highly expressed in PC tissues and cell lines. The following functional assays demonstrated that MAFG-AS1 knockdown suppressed cell proliferation and migration while promoted cell apoptosis in PC. This was similar to the finding of a former study that MAFG-AS1 boosted cell proliferation and migration in hepatocellular carcinoma [18]. Based on the above findings, we inferred MAFG-AS1 as an oncogene in PC.

Substantial literatures analyzed the role of lncRNAs in ceRNA mechanism in the development of multiple cancers [19]. As an example, lncRNA ATB was depicted as a ceRNA of CTNNB1 to bind with miR-200a [20]. MiRNAs have been recognized as the main regulators of cancer development as well. For instance, miR-511 was described to suppress breast cancer cell proliferation and invasion through targeting FGF4 [21]. In this study, we found miR-3196 could bind with MAFG-AS1. MiR-3196 was measured to be lowly-expressed in PC cell lines, and the up-regulation of miR-3196 repressed PC cell proliferative and migratory capacities, which was consistent with its function in breast cancer [22]. Moreover, many researches proved that miRNAs can bind to the 3′ UTR of mRNAs to silence these mRNAs. In this research, we assumed NFIX was the downstream target of miR-3196 and demonstrated that NFIX expression was positively regulated by MAFG-AS1 and negatively regulated by miR-3196. Existing research verified that NFIX acted as an oncogene in gastric cancer [23]. The current study discovered NFIX was highly expressed in PC cells. Besides, up-regulation of NFIX could restore the suppressive impacts of MAFG-AS1 silence on PC cell growth and migration.

Our research unveiled a ceRNA network of MAFG-AS1/miR-3196/NFIX axis in PC, highlighting MAFG-AS1 as a promising target for PC treatment. However, the deficiency of in vivo evidences is the main limitation of the current work, and this will be solved in the future. The clinical significance of MAFG-AS1 in PC needs to be further testified. Moreover, we will further focus on the reason why MAFG-AS1 is upregulated in PC and the mechanism whereby NFIX affects PC development.

## Conclusion

In conclusion, our study validated that MAFG-AS1 facilitated the progression of PC by targeting miR-3196/NFIX pathway. Importantly, targeting MAFG-AS1 might be a potential way to treat patients with PC.

## Supplementary information

**Additional file 1: Figure S1.** **a** RT-qPCR tested MAFG-AS1 expression in 65 PC tissues and adjacent non-tumor tissues. **b** ISH assay examined MAFG-AS1 staining in PC tissues and paired non-tumor tissue. Scale bar=50 μm. **P<0.01.

## Data Availability

Related data and materials could be seen in the manuscript and the supplementary files.

## Abbreviations

PC: Pancreatic cancers
MAFG-AS1: MAF bZIP transcription factor G antisense RNA 1
NFIX: Nuclear factor I X
lncRNAs: Long non-coding RNAs
mRNAs: Messenger RNAs
miRNAs: MicroRNAs
ceRNA: Competing endogenous RNA
ATCC: American Type Culture Collection
FBS: Fatal Bovine Serum
RT-qPCR: Real-time quantitative polymerase chain reaction
EdU: 5-Ethynyl-2′-deoxyuridine
DAPI: 4′,6-Diamidino-2-phenylindole
TdT: Terminal deoxynucleotidyl transferase
TUNEL: DUTP Nick-End Labeling
FISH: Fluorescence in situ hybridization
RIP: RNA immunoprecipitation
WT: Wild-type
Mut: Mutant
SD: Standard deviation
ANOVA: Analysis of Variance
