# Supplementary figures and images for: MAFG-AS1 aggravates the progression of pancreatic cancer by sponging miR-3196 to boost NFIX
Source: Cancer Cell Int. 2020 Dec 9;20:591. doi: 10.1186/s12935-020-01669-y (PMC7724861; doi:10.1186/s12935-020-01669-y)

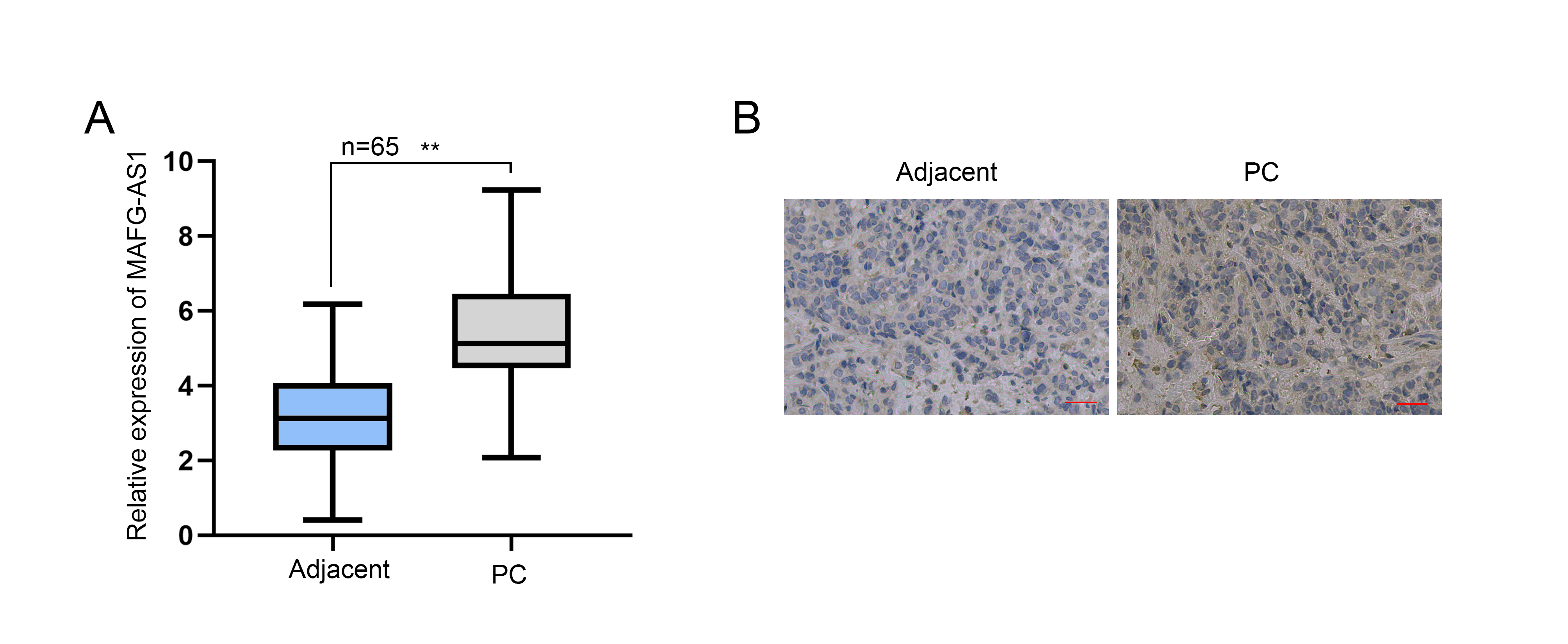

Supplement: Supplementary file 1 — Additional file 1: Figure S1. a RT-qPCR tested MAFG-AS1 expression in 65 PC tissues and adjacent non-tumor tissues. b ISH assay examined MAFG-AS1 staining in PC tissues and paired non-tumor tissue. Scale bar=50 μm. **P<0.01. [file 12935_2020_1669_MOESM1_ESM.tif]
